# Supplementary material for: Quinolinic acid metabolism may mitigate AKI to CKD transition
Source: bioRxiv. 2025 Oct 13:2025.10.10.681643. Preprint. [Version 2] doi: 10.1101/2025.10.10.681643 (PMC12632480; doi:10.1101/2025.10.10.681643)
Supplement: Supplement 1 [file media-1.pdf]

Supplementary materials- Saade and Saliba et al.

Quinolinic acid metabolism may mitigate AKI to CKD transition

Supplementary Figure S1: Folic acid (FA)-induced mouse model of AKI-to-CKD transition.

Supplementary Figure S2: Nicotinamide supplementation in the folic acid-induced AKI-to-CKD transition model.

Supplementary Figure S3: Serum creatinine and BUN levels in 72-week-old *Qprt* $^{+/+}$ (WT) vs. *Qprt* $^{+/-}$ (HT) mice.

Supplementary Figure S4: Renal injury and quinolinic and acid levels in *Haao- Kmo*-Knockout mice compared to wild-type.

Supplementary Table S1: Top differentially expressed genes in kidneys of mice administered with 0.5 g/L quinolinic acid in drinking water for 14 days.

Supplementary Table S2: Metadata for selected healthy reference (HRT) and acute kidney injury (AKI) samples from Kidney Precision Medicine Project (KPMP)

Supplementary Table S3: Pediatric chronic kidney disease (CKD) study participants characteristics

Supplementary Table S4: End stage kidney disease study participants characteristics

Supplementary Table S5: Data analysis of reduction ratios of BUN and kynurenine pathway metabolites after a single hemodialysis session in the end stage kidney disease study

Supplementary Methods

Supplementary Figure S1: Folic acid (FA)-induced mouse model of AKI-to-CKD transition. Wild type mice were injected with vehicle (n=10) or FA 250mg/kg (n=12) on day 0. Animals were sacrificed on day 14. (A) Serum creatinine, (B) BUN and (C) *Tgfb* mRNA expression in kidneys.

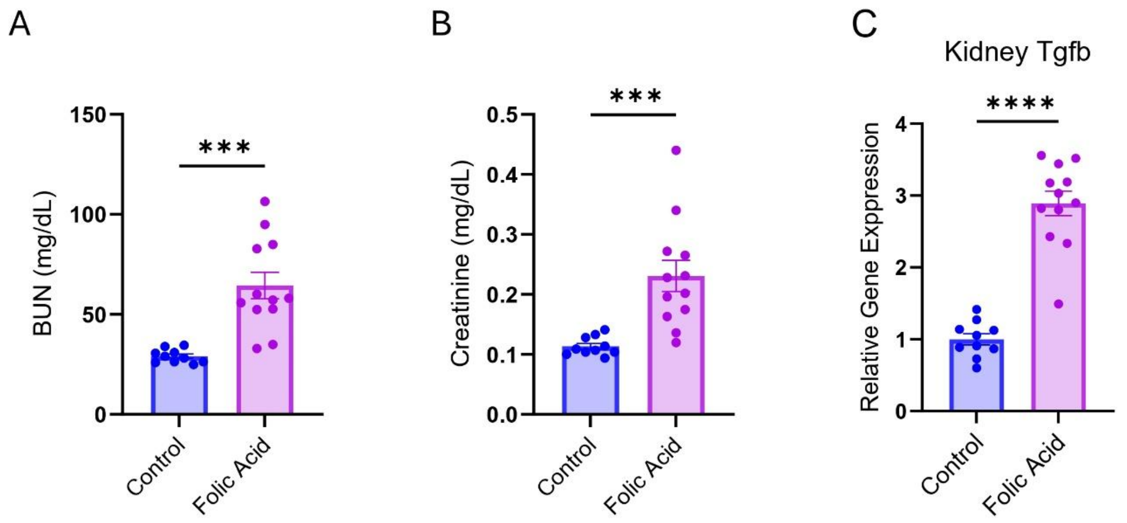

Supplementary Figure S2: Nicotinamide supplementation in the folic acid-induced AKI-to-CKD transition model. Mice were administered with 0.6% NAM in drinking water starting on day 0, injected intraperitoneally with 250 mg/kg FA on the same day and sacrificed on day 14 (n=5-8 per group). Metabolites were measured by untargeted metabolomics, including plasma nicotinamide (NAM) (**A**), creatinine (**B**), urate (**C**). Renal *Acta2* (**D**) and *Lcn2* (**E**) mRNA expression. All data are presented as mean ± SEM. Statistical analysis was performed using one-way ANOVA with post hoc testing. $p < 0.05$ (*), $p < 0.01$ (**), $p < 0.001$ (***).

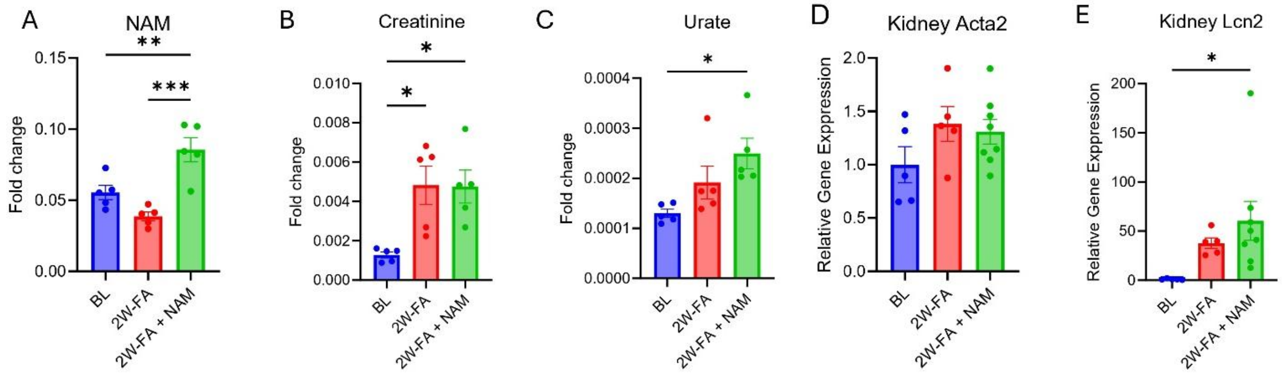

Supplementary Figure S3: Serum creatinine and BUN levels in 72-week-old *Qprt* $^{+/+}$ (WT) vs. *Qprt* $^{+/-}$ (HT) mice. - related to Figure 3 (**A**) Serum creatinine and (**B**) BUN in aged *Qprt* $^{+/+}$ (WT) vs. *Qprt* $^{+/-}$ (HT) mice (n=7-14 per group). All data are presented as mean ± SEM.

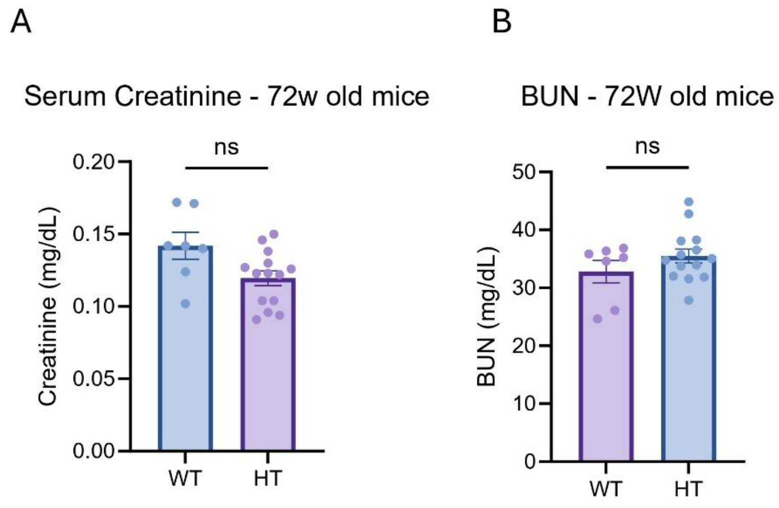

Supplementary Figure S4: Renal injury and quinolinic and acid (QA) levels in Haao- Kmo-Knockout mice compared to wild-type. (A) BUN levels, **(B)** Plasma creatinine levels**,** (**C**) plasma tryptophan **(D)** plasma kynurenine, (**E**) plasma QA, **(F)** kidney tryptophan **(G)** kidney kynurenine, (**H**) Kidney QA levels in wild-type (WT), *Haao* $^{-/-}$ (denoted "A") and *Kmo*$^{-/-}$ (denoted "K") n=4 per group C57BL/6N background. All data are presented as mean ± SEM. Statistical analysis was performed using one-way ANOVA. $p < 0.05$ (*), $p < 0.01$ (**), $p < 0.0001$ (****).

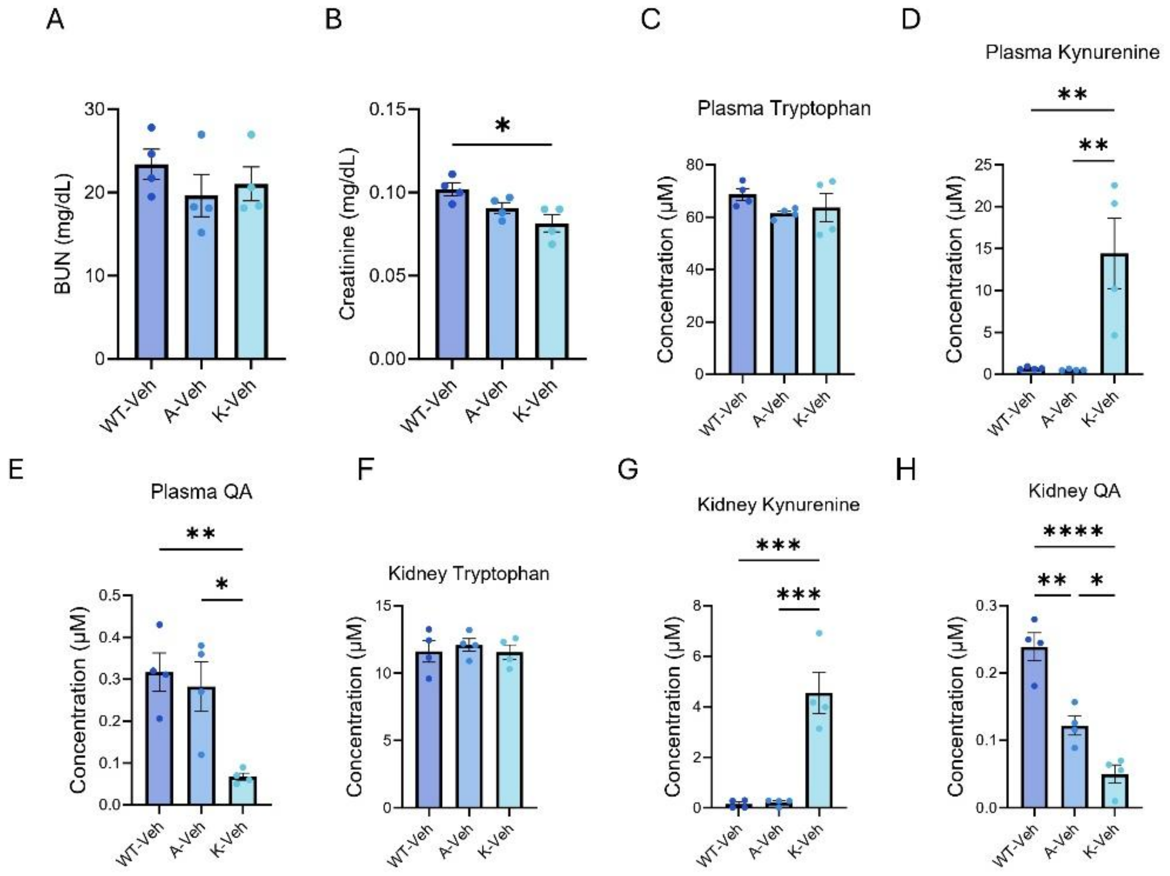

Supplementary Table S1. Top differentially expressed genes in kidneys of mice administered with 0.5 g/L quinolinic acid in drinking water for 14 days.

**Gene abbreviation**	**Gene full name**	**Log2 Fold change**	**Base -10 logarithm of p-value**
Dusp1	Dual Specificity Phosphatase 1	0.772965198	8.93287844
Gadd45g	Growth Arrest and DNA Damage Inducible Gamma	1.102903637	5.85149085
Adipoq	Adiponectin, C1Q And Collagen Domain Containing	4.594791601	4.63868612
Sgk1	Serine/threonine-protein kinase	0.611314515	4.00199938
Rbp4	Retinol Binding Protein 4	1.70827297	3.88132035
Myc	Myelocytomatosis oncogene	0.819341704	3.85776255
Rgcc	Regulator Of Cell Cycle	0.557283549	3.68874709
Orm1	Orosomucoid 1	3.29037894	3.30270992
Stra6	Signaling Receptor And Transporter Of Retinol	1.164271291	3.18095352
Angptl2	Angiopoietin-like protein 2	0.347129044	2.98379838
Bmal1	Basic Helix-Loop-Helix ARNT Like 1	-0.831754026	2.90071497
Cdk6	Cyclin-dependent kinase 6	-0.639155556	3.84606714
Serpina3i	Cyclin-dependent kinase 6	-4.092363499	3.00595597
Clock	Clock circadian regulator	-0.336020299	3.06964803

Supplementary Table S2: Metadata for selected healthy reference (HRT) and acute kidney injury (AKI) samples from Kidney Precision Medicine Project (KPMP)

Sample ID	KPMP ID	Disease Category	Age	Gender	Race	Procedure Type	Specimen Type	Significant Past Medical History
S-2008-006028	163-1	HRT	N/A	N/A	N/A	Kidney Biopsy	Kidney Cortex	available on request
S-2008-007631	164-1	HRT	N/A	N/A	N/A	Kidney Biopsy	Kidney Cortex	available on request
S-2108-005528	165-12	HRT	58	Female	White	Kidney Biopsy	Kidney Cortex	available on request
S-2109-023337	165-10	HRT	57	Female	White	Kidney Biopsy	Kidney Cortex	available on request
S-2111-008287	163-7	HRT	72	Female	White	Kidney Biopsy	Kidney Cortex	available on request
S-2111-008311	164-12	HRT	37	Female	White	Kidney Biopsy	Kidney Cortex	available on request
S-2112-014329	164-14	HRT	48	Male	White	Kidney Biopsy	Kidney Cortex	available on request
S-2111-008287	164-16	HRT	59	Male	White	Kidney Biopsy	Kidney Cortex	available on request
S-2006-001713	30-10868	AKI	55	Male	White	Kidney Biopsy	Kidney Cortex	available on request
S-2006-001760	30-11084	AKI	60	Female	White	Kidney Biopsy	Kidney Cortex	available on request
S-2006-001901	32-10456	AKI	41	Male	Black or African-American	Kidney Biopsy	Kidney Cortex	available on request

S-2006-001948	30-11101	AKI	46	Male	Black or African-American	Kidney Biopsy	Kidney Cortex	available on request
S-2006-001995	32-10333	AKI	46	Male	Asian	Kidney Biopsy	Kidney Cortex	available on request
S-2006-002396	33-10376	AKI	N/A	N/A	N/A	Kidney Biopsy	Kidney Cortex	available on request
S-2006-002492	33-10331	AKI	30	Female	African American	Kidney Biopsy	Kidney Cortex	available on request
S-2006-004487	34-10209	AKI	53	Male	White	Kidney Biopsy	Kidney Cortex	available on request
S-2101-003289	30-11093	AKI	20	Male	White	Kidney Biopsy	Kidney Cortex	available on request
S-2101-003336	30-11081	AKI	73	Female	White	Kidney Biopsy	Kidney Cortex	available on request
S-2102-003410	34-10240	AKI	60	Female	White	Kidney Biopsy	Kidney Cortex	available on request
S-2102-003504	34-10393	AKI	31	Male	White	Kidney Biopsy	Kidney Cortex	available on request
S-2109-011411	32-10346	AKI	44	Male	Black or African-American	Kidney Biopsy	Kidney Cortex	available on request
S-2109-019203	34-10579	AKI	64	Female	Black or African-American	Kidney Biopsy	Kidney Cortex	available on request

S-2109-019250	34-10666	AKI	48	Male	Black or African-American	Kidney Biopsy	Kidney Cortex	available on request
S-2209-009415	34-10730	AKI	24	Male	White	Kidney Biopsy	Kidney Cortex	available on request
S-2304-011687	34-10889	AKI	49	Male	Other	Kidney Biopsy	Kidney Cortex	available on request
S-2309-028497	34-11066	AKI	40	Male	Black or African-American	Kidney Biopsy	Kidney Cortex	available on request
S-2401-002293	933-10372	AKI	66	Male	White	Kidney Biopsy	Kidney Cortex	available on request

Supplementary Table S3: Pediatric chronic kidney disease (CKD) study participants characteristics (control n=36 and CKD n=72)

Patient Characteristics	Control	CKD
**Age (mean)**	11.2	12.6
**Female gender (%)**	50%	45.80%
**Race**		
American Indian	0%	0%
Asian	2.70%	8.45%
African American	22.20%	29.57%
Hispanic	16.60%	9.85%
White	58%	52.11%
Hawaiian	0%	0%

Supplementary Table S4: End stage kidney disease study participants characteristics (n = 29).

Age (Year)	Gender	Pre_WT (Kg)	Post-WT (Kg)	URR	Pre_BUN (mg/dL)	Post_BUN (mg/dL)	Creatinine Serum (mg/dL)	Pre_ Quinolinic acid (µM)	Post_ Quinolinic acid (µM)
64	M	90.50	88.10	78.0	59.0	13.0	6.9	2.346811	0.03483
82	F	73.50	71.60	81.4	43.0	8.0	6.1	11.10638	1.094067
72	F	62.10	59.70	90.3	31.0	3.0	5.6	3.7689	0.023947
43	F	81.40	77.10	82.4	51.0	9.0	7.6	7.892067	0.466936
49	M	84.20	80.00	73.2	56.0	15.0	10.6	4.298414	0.567978
51	M	75.10	73.00	77.0	61.0	14.0	9.9	7.196925	0.144499
56	F	61.30	60.20	85.0	60.0	9.0	9.1	4.104453	0.02988
36	M	94.80	92.40	70.0	80.0	24.0	14.6	15.22459	4.155645
67	F	75.70	73.50	74.5	51.0	13.0	9.1	10.69891	0.470443
56	M	61.50	57.20	80.0	25.0	5.0	7.8	9.606912	0.389974
59	F	74.80	71.40	86.7	60.0	8.0	7.8	5.59861	0.022882
58	M	52.30	50.30	89.4	66.0	7.0	11.9	4.251141	0.045559
47	F	85.90	81.10	84.0	71.0	11.0	7.7	4.16625	0.130175
25	F	101.50	97.90	74.5	51.0	13.0	11.9	7.463583	0.361765
74	M	84.70	82.50	86.0	43.0	6.0	7.4	5.453344	0.197385
73	M	70.50	68.10	81.7	71.0	13.0	10.4	8.040016	0.057851
63	F	76.30	73.00	88.0	92.0	42.0	7.5	5.23093	0.136084
68	F	62.60	61.10	88.2	34.0	4.0	7.3	3.941061	0.047809
60	F	93.80	90.30	82.0	57.0	10.0	9.8	9.578379	0.451747
53	F	88.50	85.50	80.0	36.0	7.0	7.4	10.43136	0.907096
54	F	94.30	91.60	78.0	52.0	10.0	9.01	11.29416	1.023004
63	F	80.60	78.80	91.0	47.0	4.0	7.9	8.134879	0.26983
69	F	58.50	58.30	88.0	35.0	4.0	4.9	12.18456	0.288326
32	F	112.40	110.60	78.0	76.0	16.0	9.1	10.57339	0.81875
52	F	96.20	93.00	75.0	48.0	9.0	10.1	4.757922	0.204565
59	M	74.90	72.90	78.0	67.0	9.0	8.5	6.706208	0.525585

68	M	76.90	73.80	80.0	83.0	13.0	11.5	3.815608	0.107667
55	M	158.50	153.80	70.0	62.0	20.0	8.7	6.831568	0.69637
47	F	45.40	44.00	88.9	45.0	5.0	6.5	4.286252	0.063804

WT, weight; URR, urea reduction rate, BUN, blood urea nitrogen

Supplementary Table S5: Data analysis of reduction ratios of BUN and kynurenine pathway metabolites after a single hemodialysis session in the end stage kidney disease study

	**URR**	**QARR**	**3HKRR**	**KYNRR**	**3HAARR**	**TRP RR**	**xant RR**	**KYNARR**
**Number of values**	29	29	28	28	28	28	28	28
**Minimum**	70.00	72.70	18.56	9.788	48.59	-104.1	23.72	10.56
**Maximum**	91.00	99.59	97.85	94.52	91.44	68.28	70.33	72.15
**Range**	21.00	26.89	79.29	84.73	42.85	172.4	46.61	61.60
**Mean**	81.35	94.84	85.10	56.61	71.27	-5.691	46.28	43.37
**Std. Deviation**	6.064	5.440	16.46	16.21	9.837	37.05	13.48	15.00
**Std. Error of Mean**	1.126	1.010	3.111	3.064	1.859	7.001	2.548	2.834
**P-value (two tailed t test)**	5.1913E-19	1.22487E-15	3E-12	2E-09	1.6E-11	0.51887	6.8E-09	0.002115748

Reduction ratio (RR) represents the percent decrease in each analyte post-dialysis. QA, quinolinic acid; 3HK, 3-hydroxykynurenine; KYN, kynurenine; 3HAA, 3-hydroxyanthranilic acid; Trp, tryptophan; Xant, xanthurenic acid; KYNA, kynurenic acid; BUN, blood urea nitrogen.

Supplementary Methods

RNA sequencing. RNA quality was assessed using the Agilent Tapestation 4200, and only samples with an RNA Integrity Number (RIN) ≥ 8 were included for sequencing. RNA concentration was measured with a Qubit® 4.0 Fluorimeter (ThermoFisher). One microgram of DNase-treated total RNA was used for library preparation with the TruSeq Stranded mRNA Library Prep Kit (Illumina). Poly(A) RNA was purified and fragmented, followed by strand-specific cDNA synthesis. The resulting cDNA was A-tailed, ligated to indexed adapters. After adapter ligation, samples are PCR amplified and purified with AmpureXP beads, then validated again on the Agilent Tapestation 4200. Before being normalized and pooled, samples are quantified by Qubit then run on the Illumina NextSeq 2000 on a P2-100 flowcell. Raw sequencing data were processed with bcl2fastq to generate FASTQ files. Gene-level read counts were obtained using featureCounts, and differential expression analysis was performed with edgeR, using the mm10 reference genome.

Supplementary Figure S2. Eight-week-old WT C57BL/6J mice were used for this study. All experimental procedures were approved by the Institutional Animal Care and Use Committee at the University of Texas Southwestern Medical Center and conducted in accordance with the NIH Guide for the Care and Use of Laboratory Animals. Animals were randomly assigned into three groups. Control group (n = 5): Mice received standard drinking water for 14 days and were injected intraperitoneally with folic acid (FA, 250 mg/kg) on day 0. Animals were sacrificed on day 14. NAM-treated group (n = 8): Mice were administered 0.6% nicotinamide (NAM) in drinking water starting on day 0 and were injected intraperitoneally with FA (250 mg/kg) on day 0. Drinking water was exchanged every other day. Animals were sacrificed on day 14.

qPCR primers list

Mouse_*Qprt:*

F_ GAAAGACAACCATGTAGTGGCGG; R_ GGCTGCTACATTCCACCTCTAC

Mouse_*Col3a1:*

F_GACCAAAAGGTGATGCTGGACAG; R_CAAGACCTCGTGCTCCAGTTAG

Mouse_*Tgfb*:

F_TGATACGCCTGAGTGGCTGTCT; R_CACAAGAGCAGTGAGCGCTGAA

Mouse_*Acta2:*

F_TGCTGACAGAGGCACCACTGAA; R_CAGTTGTACGTCCAGAGGCATAG

Mouse_*Lcn2:*

F_ ATGTCACCTCCATCCTGGTCAG; R_GCCACTTGCACATTGTAGCTCTG

Mouse_*Nfkb:*

F_ CCCCCTGGTGGAGAACTTTG; R_ GCTGCTTCATGTCCCCTTGT

Mouse_*Pdgfra:*

F_ CATGCGGGTGGACTCTGATA; R_ CCGAAGTCTGTGAGCTGTGT

Mouse_*Gapdh:*

F_TGTGTCCGTCGTGGATCTGA; R_CCTGCTTCACCACCTTCTTGAT

Mouse_*Actin:*

F_CATTGCTGACAGGATGCAGAAGG; R_TGCTGGAAGGTGGACAGTGAGG
